# Polycrystalline Diamond Coating on Orthopedic Implants: Realization and Role of Surface Topology and Chemistry in Adsorption of Proteins and Cell Proliferation

**DOI:** 10.1021/acsami.2c10121

**Published:** 2022-09-22

**Authors:** Justas Zalieckas, Ivan R. Mondragon, Paulius Pobedinskas, Arne S. Kristoffersen, Samih Mohamed-Ahmed, Cecilie Gjerde, Paul J. Høl, Geir Hallan, Ove N. Furnes, Mihaela Roxana Cimpan, Ken Haenen, Bodil Holst, Martin M. Greve

**Affiliations:** † Department of Physics and Technology, University of Bergen, Allegaten 55, 5007 Bergen, Norway; ‡ Department for Clinical Dentistry, University of Bergen, Årstadveien 19, 5009 Bergen, Norway; § Institute for Materials Research (IMO), Hasselt University, Wetenschapspark 1, 3590 Diepenbeek, Belgium; ∥ IMOMEC, Interuniversity MicroElectronics Center (IMEC) vzw, Wetenschapspark 1, 3590 Diepenbeek, Belgium; ⊥ Department of Orthopaedic Surgery, Haukeland University Hospital, Jonas Lies vei 65, 5021 Bergen, Norway; # Department of Clinical Medicine, University of Bergen, Jonas Lies vei 87, 5021 Bergen, Norway

**Keywords:** diamond, surface wave plasma, orthopedic implants, acetabular shell, collagen, albumin, cell proliferation

## Abstract

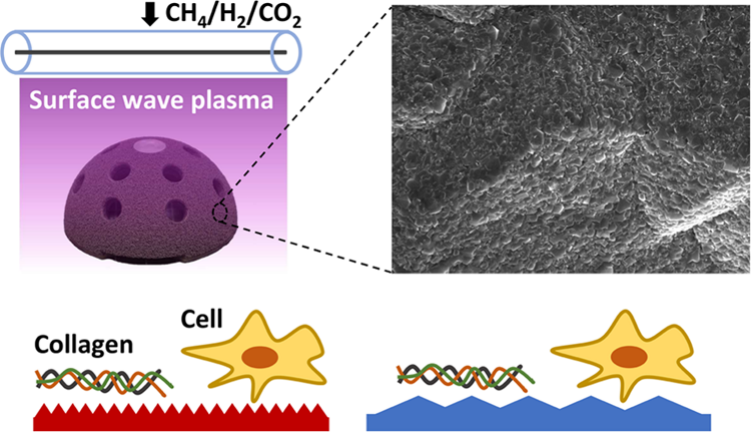

Polycrystalline diamond has the potential to improve the osseointegration of orthopedic implants compared to conventional materials such as titanium. However, despite the excellent biocompatibility and superior mechanical properties, the major challenge of using diamond for implants, such as those used for hip arthroplasty, is the limitation of microwave plasma chemical vapor deposition (CVD) techniques to synthesize diamond on complex-shaped objects. Here, for the first time, we demonstrate diamond growth on titanium acetabular shells using the surface wave plasma CVD method. Polycrystalline diamond coatings were synthesized at low temperatures (∼400 °C) on three types of acetabular shells with different surface structures and porosities. We achieved the growth of diamond on highly porous surfaces designed to mimic the structure of the trabecular bone and improve osseointegration. Biocompatibility was investigated on nanocrystalline diamond (NCD) and ultrananocrystalline diamond (UNCD) coatings terminated either with hydrogen or oxygen. To understand the role of diamond surface topology and chemistry in the attachment and proliferation of mammalian cells, we investigated the adsorption of extracellular matrix proteins and monitored the metabolic activity of fibroblasts, osteoblasts, and bone-marrow-derived mesenchymal stem cells (MSCs). The interaction of bovine serum albumin and type I collagen with the diamond surfaces was investigated by confocal fluorescence lifetime imaging microscopy (FLIM). We found that the proliferation of osteogenic cells was better on hydrogen-terminated UNCD than on the oxygen-terminated counterpart. These findings correlated with the behavior of collagen on diamond substrates observed by FLIM. Hydrogen-terminated UNCD provided better adhesion and proliferation of osteogenic cells, compared to titanium, while the growth of fibroblasts was poorest on hydrogen-terminated NCD and MSCs behaved similarly on all tested surfaces. These results open new opportunities for application of diamond coatings on orthopedic implants to further improve bone fixation and osseointegration.

## 1 Introduction

Conventionally used materials for medical implants are not ideal and come with limitations, which can lead to complications or even to a worst outcome: a revision surgery. The ideal biomaterial is expected to promote cellular growth, inhibit bacterial adhesion, and have excellent tribological properties. One of the most widely used materials for orthopedic implants, such as for a hip replacement, is titanium and its alloys. However, the lifetime of such implants is limited and typically ranges from 5 to 25 years. Revision surgeries are more complex, take longer time, are more costly, and have a greater risk of complications. Typically, 4–5% of people who receive a hip implant may require a revision surgery within 10 years and 15% of patients need a revision surgery within 20 years.^1^ The most common reasons for the failure of a titanium prosthesis are aseptic loosening and bacterial infection, both of which are directly related to the surface properties of titanium. This can be attributed to the fact that the surface of titanium has limited bioactivity and lacks antibacterial properties.^2^ Therefore, much effort has been taken to improve the performance of titanium by various modifications and structuring of the surface or by application of coatings.^3^ Conventional materials suggested as coatings on titanium for enhanced biocompatibility include hydroxyapatite, bioactive glass, biphasic calcium phosphate, and TiN. However, the stability, adhesion, and degradation performance of these coatings are still challenging.^4,5^ The drawbacks and limited performance can be partly attributed to the quality and coverage of coatings, which has a direct effect on responses, such as initial cellular adhesion to a substrate, subsequent growth and proliferation, and bioactivity of the material.^6^

One of the most promising candidates to address the drawbacks of state-of-the-art coatings is diamond. Diamond as a coating on orthopedic implants provides several solutions due to its unique properties, including wear resistance, high biocompatibility, corrosion resistance, chemical inertness, and high adhesion to titanium. All of these properties potentially make diamond an ideal coating for orthopedic implants, overcoming the shortcomings of currently used solutions. It has been demonstrated that diamond coatings promote osteoblast adhesion,^7−9^ show antimicrobial properties,^10,11^ and have a high degree of biocompatibility.^12^ Moreover, diamond showed high tribological performance for coated femoral heads in a wear simulator,^13^ and it has high potential of being used also for wear-intense applications.

The standard method used for the growth of diamond on the titanium substrate is chemical vapor deposition (CVD). Depending on the type of the energy source used to activate carbon-containing gas, there are two most widely used CVD techniques: hot filament (HF) CVD and microwave plasma-enhanced (MWPE) CVD with diamond films, typically grown at substrate temperatures ranging from 500 to 1000 °C.^14^ In HFCVD reactors, gasses are activated by heated tungsten wires (filaments), while in the MWPECVD reactors, microwave radiation is used as an energy source to generate a gas discharge. The HFCVD can achieve large-area CVD but suffers from filament instability and contamination of the growing diamond film.^15,16^ Therefore, for the growth of high-purity diamond films, typically, resonant-cavity MWPECVD systems operating at 2.45 GHz frequency are used. The deposition area of these systems is limited up to approximately 30 cm^2^^17^ by the gas discharge shape and size, which is roughly half the wavelength at a given frequency. These restrictions and the planar-type nature of the above-listed techniques have limited the size and shape of the objects, which can be used for the synthesis of diamond. The current state-of-the-art is Rifai et al.’s^18^ demonstration of diamond deposition on additively manufactured hollow 3 × 3 × 3 mm^3^ titanium cubes using the MWPECVD method, followed by the investigation of diamond synthesis on samples up to 8 × 8 × 3 mm^3^ using a protective Faraday cage.^19^ Maru et al.^13^ used the HFCVD method to deposit diamond on femoral heads 28 mm in diameter but did not study film uniformity.

As an alternative to HFCVD and resonant-cavity MWPECVD, either a distributed antenna array (DAA)^20^ or a surface wave plasma (SWP)^21,22^ CVD system could be used for diamond synthesis even at temperatures below 100 °C.^23^ These two techniques operate at low pressures (<2 mbar) and yield larger gas discharge volumes compared to HFCVD and MWPECVD methods, which is beneficial for diamond growth on complex-shaped objects. The latest demonstrations come from Dekkar et al.,^24^ showing diamond growth on a cylindrical-shaped titanium implant of 6.3 mm height with the DAA CVD system, and Varga et al.,^25^ achieving nonuniform growth on copper rods 2.5 mm in diameter and approximately 30 mm in length using SWP CVD. Therefore, there is a need to investigate diamond synthesis on larger than the above-mentioned objects to facilitate the use of diamond-based materials for orthopedic implants such as a hip replacement.

Surface topology and chemistry of diamond coatings play an important role in adsorption of proteins and cell proliferation and viability. Alcaide et al.^26^ showed that the topology and doping of polycrystalline diamond films alter the adsorption of serum proteins and can influence the resistance of fibroblast adhesion and proliferation. Cytotoxicity evaluation of fibroblasts on diamond coatings showed no induced cytotoxic response.^27^ Liskova et al.^28^ found that osteoblasts exhibited a higher growth rate on oxygen-terminated diamond films compared to hydrogen-terminated counterparts. Furthermore, they found that the oxygen-terminated surface supports the deposition of extracellular matrix (ECM) proteins. A recent study from Rifai^29^ shows that polycrystalline diamond promotes expression of adhesion proteins and that surface topology can guide the proliferation of osteoblasts. As suggested by Fong et al.,^6^ mesenchymal stem cells (MSCs) can be integrated on diamond coatings to improve osteoconductive properties of implants; however, the literature within the field reports inconsistent findings on MSC adhesion and proliferation on diamond.^30^ Therefore, it is important to study multiple cell types to gain a better understanding of cell adhesion and proliferation on diamond substrates. Furthermore, since cells adhere to a surface via focal adhesion points interacting with ECM, investigations of adsorption of fibrous ECM proteins on hydrogen- and oxygen-terminated diamond surfaces should complement the above-mentioned studies.

Here, for the first time, we use the SWP CVD method to synthesize diamond on complex-shaped orthopedic implants, namely, titanium acetabular shells, taken from patients after revision hip replacement surgeries. The biocompatibility and properties of the films were investigated on two types of coatings, nanocrystalline diamond (NCD) and ultrananocrystalline diamond (UNCD), deposited on silicon wafers and titanium hemispheres designed to mimic the shape of the acetabular shells. We evaluated the metabolic activity of fibroblasts, osteoblasts, and MSCs on NCD and UNCD surfaces terminated with hydrogen and oxygen, showing that proliferation and viability of MSCs are best on hydrogen-terminated UNCD. Furthermore, we show that hydrogen-terminated UNCD provides better adhesion and proliferation for osteogenic cells, compared to the titanium substrate. Lastly, for the first time, we complement the biocompatibility assessment of the cells with the investigation of adsorption of blood and ECM proteins on the diamond surface. We show that type I collagen adsorption and behavior observed by confocal fluorescence lifetime imaging microscopy (FLIM) correlate with the proliferation of MSCs and osteogenic cells on hydrogen- and oxygen-terminated UNCD.

## 2 Experimental Section

### 2.1 Synthesis of Polycrystalline Diamond

#### 2.1.1 Sample Preparation

Polycrystalline diamond was synthesized on three hemispherical acetabular shells taken from patients after revision hip replacement surgeries and donated by the Haukeland University Hospital in Bergen, Norway. The bulk of all acetabular shells was made from the Ti–6Al–4V alloy with the following types of backing materials: (i) porous metal made from the trabecular-type tantalum material, referred to as “TRABECULAR” (cat. no. T/TA 6202-58-20, Trabecular Metal Modular, Zimmer, *d* = 58 mm), (ii) fiber mesh made from commercially pure (CP) titanium, referred to as “M-MESH” (cat. no. T6610-54-02, Harris Galante II, Zimmer, *d* = 54 mm), and (iii) arc-deposited plasma sprayed CP titanium, referred to as “TRIDENT” (cat. no. 500-01-58F, Trident, Stryker, *d* = 58 mm). The residual bone content on acetabular shells was mechanically brushed away in warm water (50–70 °C), followed by ultrasonication in acetone for 30 min. The uniformity of diamond coatings was investigated on hemispheres 40 and 60 mm in diameter machined from the Ti–6Al–4V alloy and polished using a high-capacity finisher (Radiance 50, Schmidts Polérmedel).

#### 2.1.2 Diamond Coating

Prior to deposition, all samples were exposed for 3 min ex situ to reactive oxygen gas plasma to achieve a good nanodiamond (ND) seeding density using the same process conditions as in refs (31, 32) The acetabular shells and titanium hemispheres were seeded by pouring a water-based colloidal solution of ultradispersed ND particles (5–7 nm in diameter) over them and rinsing them afterward with deionized water. The ND colloid was prepared as in ref^33^ from detonation ND powder provided by the NanoCarbon Institute Co., Ltd. Silicon substrates (polished 4 in. wafers) were seeded with the same ND suspension via drop-casting and subsequent spin-drying as detailed in ref (33). Polycrystalline diamond samples were prepared by SWP CVD (W&L Coating Systems, TruDi MWPECVD System), keeping all samples 2.5 cm away from the linear antenna (LA). A CVD gas mixture consisting of 2% methane (CH_4_), 6% carbon dioxide (CO_2_), and 92% hydrogen (H_2_) was used to grow NCD films, while UNCD films were synthesized using 8% CH_4_, 6% CO_2_, and 86% H_2_. Carbon dioxide was added to ensure effective etching of sp^2^ carbon phases at low temperatures.^34,35^ The NCD and UNCD films were grown for 22 and 10 h, respectively. Both types, NCD and UNCD films, were deposited on titanium hemispheres and silicon wafers, while only NCD was synthesized on acetabular shells. The temperature of the samples 2.5 cm away from the LA was measured to be ∼400 °C. The microwave power, gas pressure, and total gas flow were 2800 W, 0.22 mbar, and 150 sccm, respectively.

#### 2.1.3 Surface Treatment

The silicon wafers were cut into smaller samples and divided into two batches. Samples from the first batch were terminated with hydrogen by exposing them to hydrogen plasma inside an in-house built MWPECVD reactor for 10 min at 600–700 °C and 50 mbar, keeping the microwave power at 1400 W. A reactive ion etcher Plasmatherm 790+ was used to terminate the samples from the second batch with oxygen. The samples were placed on a grounded holder and exposed to oxygen plasma at 0.133 mbar pressure and room temperature for 2 min with no added bias, keeping the power at 100 W.

### 2.2 Material Characterization

The surface morphology of the polycrystalline diamond films was examined with Raith e-Line and Zeiss SUPRA 55VP scanning electron microscopes (SEM), using in-lens secondary electron detectors and an acceleration voltage of 10 kV. Surface topology and roughness were investigated with a Bruker Dimension Icon atomic force microscope (AFM), employing peak force tapping mode (ScanAsyst) with a ScanAsyst-Air probe (Bruker). The film thickness of flat and curved surfaces was measured using a spectral reflectance technique with a Filmetrics F10-RT reflectometer. Curved surfaces to the first-order approximation were considered as flat. Since the formation of the TiC interface during the CVD process typically yields good adhesion between the diamond and titanium substrate,^36^ adhesion strength of diamond films was not investigated in this study.

The composition of the diamond films was examined by Raman spectroscopy, measuring spectra in the 1000–2000 cm^–1^ range with a HORIBA LabRAM 800 HR spectrometer working in confocal mode and using a 488 nm wavelength Ar laser as an excitation source. The chemical composition of the coated surfaces was investigated with an Axis Ultra DLD (Kratos Analytical) X-ray photoelectron spectrometer (XPS). High-resolution XPS spectra were taken by probing 700 × 300 μm^2^ areas using a monochromatic Al Kα X-ray source operating at 10 kV and 10 mA. Survey and regional scans were acquired with a pass energy of 160 and 20 eV, respectively. The step size was set to 1 eV for the survey and 0.1 eV for regional scans. The reported spectra were charge-corrected with reference to adventitious carbon (C 1s peak at 284.8 eV). Acquired data were analyzed using CasaXPS (Casa Software Ltd.).

The surface wettability of titanium and diamond samples was characterized with a video-based optical contact angle measurement system OCA20 LHT (Dataphysics) by measuring the static water contact angle. The contact angles were measured 3 times for each surface at room temperature by gently depositing water droplets having a volume of 3 μL. The measurements on diamond surfaces were done within 15 min after the plasma treatment (see Section 2.1.3) to avoid surface contamination by hydrocarbons and change of wetting angles.^31^

### 2.3 Adsorption of Proteins

#### 2.3.1 Sample Preparation

Bovine serum albumin (BSA) and type I collagen (COL) from bovine skin, both labeled with fluorescein isothiocyanate (FITC), were purchased from Sigma-Aldrich. Silicon samples with hydrogenated and oxygenated NCD and UNCD were immersed in BSA (1 mg/mL in 10 mM Tris buffer at pH 7.4) and COL (1 mg/mL in 0.01 M acetic acid at pH 5.0) solutions for 1 h at room temperature. Subsequently, samples were rinsed three times and submerged in 10 mM Tris buffer at pH 7.4 prior to the fluorescence lifetime measurements.

#### 2.3.2 Fluorescence Lifetime Imaging Microscopy (FLIM)

Fluorescence lifetime data of BSA^FITC^ and COL^FITC^ conjugates were obtained using time-correlated single-photon counting (TCSPC). A Ti/sapphire laser (Coherent Chameleon Ultra) tuned to 900 nm wavelength, generating femtosecond pulses (pulse width 140 fs) at an 80 MHz repetition rate (12.5 ns between each pulse), was used for two-photon excitation of the samples. Excitation light was guided to a confocal inverted microscope (Leica TCS SP5) and focused by a water immersion objective (NA = 1.2). The samples were scanned at a line frequency of 400 Hz, and fluorescence of FITC was detected by a built-in photomultiplier tube (PMT) in a range of 500–700 nm. Line, frame, and pixel clock signals were generated and synchronized by a Hamamatsu R3310-02 PMT detector and linked via a TCSPC imaging module (SPC-830, Becker-Hickl) to generate fluorescence lifetime data. The fluorescence lifetime data for each sample was collected by scanning a 110 × 110 μm^2^ area with a spatial resolution of 128 × 128 pixels. The collected photons for each pixel were stored as a histogram (decay trace). We used a biexponential decay model convoluted with the instrument response function (IRF) to represent the data of each pixel. To increase the signal-to-noise ratio, for each pixel, 8 × 8 decay traces of the neighboring pixels were summed and the fluorescence lifetimes (τ_1_ and τ_2_) for the central pixel were obtained from the maximum-likelihood fit to the summed decay trace using SPCImage software, hence obtaining a decay matrix (128 × 128 pixels) for each tested sample.

### 2.4 Cell Growth on Diamond-Coated Substrates

#### 2.4.1 Cells and Cultivation

Human bone-marrow-derived mesenchymal stem cells (BMSCs) were isolated from two donors, a 53 year old male (BMSCm) and a 59 year old female (BMSCb), under ethical approval from the Regional Committee for Medical and Health Research Ethics in Norway (approval number: REK vest 7199). BMSCs were characterized based on the expression of a set of cell surface markers (CD34, CD45, CD73, CD90, CD105, and HLA-DR). BMSCs were cultured at a seeding density of 5 × 10^3^ cells/cm^2^ using the culture medium Minimum Essential Medium-α modification (αMEM, Thermo Fisher Scientific) supplemented with 10% fetal bovine serum (FBS, Sigma-Aldrich) and 1% antibiotics (penicillin/streptomycin, Sigma-Aldrich). Human primary lung fibroblasts (Innoprot) were cultured in fibroblast medium (Innoprot) at a seeding density of 6 × 10^3^ cells/cm^2^. The osteosarcoma cell line Saos-2 (DSMZ) was cultured at a seeding density of 1.2 × 10^4^ cells/cm^2^ in McCoy’s 5a medium (Thermo Fisher Scientific) supplemented with 15% FBS, GlutaMAX (Thermo Fisher Scientific), and 1% antibiotics. All cells were maintained in a humidified incubator with 5% CO_2_ at 37 °C. The medium was changed twice a week, and cells were subcultured when reaching 70–80% confluency. For experiments, BMSCs and fibroblasts were used at passages 3–6 while Saos-2 at passages 6–9.

#### 2.4.2 Cell Viability/Proliferation Assay

Diamond-coated silicon wafers were cut in 1.8 × 4.5 cm^2^ strips and surface-treated as described in Section 2.1.3. Titanium sheet 0.52 mm in thickness (TI010450/10, GoodFellow) was polished as described in Section 2.1.1 and cut into 1.8 × 5.0 cm^2^ strips. The strips were ultrasonicated in acetone for 30 min and then immersed in 70% ethanol for 10 min and air-dried inside a laminar flow hood. The sterile strips were stuck to the bottom of a black, bottomless 96-well plate (ProPlate MP, Grace Bio-Labs). The wells were rinsed twice with sterile water and allowed to air-dry while preparing cell suspensions in culture medium. Before seeding, cell suspensions were mixed 1:1 with a medium containing 2× RealTime-Glo MT cell viability assay (Promega) following the manufacturer’s instructions. Cells were seeded at the following densities in duplicate wells: 9350, 7800, and 12 500 cells/cm^2^ for BMSCs, fibroblasts, and Saos-2, respectively. Upon cell seeding, luminescence was measured at different time points (0, 1, 2, 4, 8, 24, and 48 h) using a microplate reader (SkanIt, Thermo Fisher Scientific) equipped with a temperature control module (37 °C). Three independent experimental repetitions were performed for each cell line. The data shown are normalized to luminescence at time 0 h.

#### 2.4.3 Immunostaining and Fluorescence Microscopy

Titanium sheet and diamond-coated silicon wafers were cut into 2.2 × 2.2 cm^2^ squares. The diamond films were terminated with hydrogen and oxygen as described in Section 2.1.3. The substrates were sterilized in 70% ethanol for 10 min and air-dried inside a laminar flow hood. The substrates were adhered to the bottom of a reusable eight-well silicon insert (flexiPERM^R^, Heraeus Instruments). Cells were seeded at the densities stated in Section 2.4.1 and cultured for 5 days. The medium was changed every second day, and on day 5, cells were fixed with 4% paraformaldehyde for 15 min. Cells were then permeabilized with 0.2% Triton X-100, blocked with 4% BSA/4% FBS, and incubated overnight at 8 °C with mouse antivinculin antibody (clone hVIN-1, Sigma-Aldrich). The antivinculin antibody was detected with goat antimouse antibody-AlexaFluor 488 (Thermo Fisher Scientific). Cells were counter-stained with DAPI and Phalloidin-Atto 565 (Sigma-Aldrich). Finally, substrates containing the stained cells were mounted on #1.5 glass coverslips using Mowiol^R^ (Sigma-Aldrich) mounting media. Specimens were imaged using a TCS SP8 confocal microscope (Leica Microsystems) equipped with hybrid detectors, white and blue diode lasers, and a 40× immersion objective (NA = 1.1). Whole volume images of the cells were acquired with a *z*-step of 0.5 μm.

#### 2.4.4 Image Analysis

Nucleus area and nucleus aspect ratio were used to indirectly analyze cell attachment, shape, and expansion. The larger nucleus area leads to the larger cell’s cytoskeleton expansion and thus better attachment. Concerning the aspect ratio, a value of 1 indicates a perfect circle while values larger than 1 describe an elongated circle (oval shape). The shape of the nucleus often correlates with the overall shape of the cell,^37−40^ which is influenced by its interaction with the substrate. Adequate cell attachment and uniform expansion lead to nucleus aspect ratios close to 1. Thus, the larger the value for the nucleus aspect ratio, the poorer the attachment to the substrate. The open-source software Fiji^41^ was used to quantify the number of cells, nucleus area, and nuclei aspect ratio at day 5 of culture onto the different substrates. Automated analysis was performed on the DAPI channel (nuclei staining) as follows: images were processed to obtain a *z*-projection based on the maximum intensity, global thresholding applied for binarization (value set to 50), holes filled, and the images segmented using a watershed algorithm. Finally, particles were counted and analyzed using the Analyze Particles tool with a minimum particle size of 120 μm^2^. A total of 10 images (*z*-projections) were analyzed for each cell type/substrate combination, except for fibroblasts plated on hydrogenated UNCD, where 20 images were used due to the low cell number. The total number of cells analyzed was specified in the respective plots and was higher than 250, except for fibroblasts.

#### 2.4.5 Statistical Analysis

The IBM SPSS Statistics v27 was used to obtain descriptive statistics data, perform normality analysis (Kolmogorov–Smirnov and Shapiro–Wilk tests) and generate boxplots. A one-way ANOVA with a Bonferroni post-hoc test was performed on normally distributed samples. Non-normal data (fibroblasts on hydrogenated substrates) were analyzed with the Kruskal–Wallis test for pairwise comparisons. The significance level is *p* ≤ 0.05.

## 3 Results and Discussion

We synthesized polycrystalline diamond on acetabular shells with three different surface structures and porosities and investigated diamond films properties on titanium hemispheres, machined to mimic the shape of the shells. The role of surface topology and chemistry of diamond in adsorption of proteins is studied by time-resolved fluorescence microscopy based on excited state lifetimes of FITC conjugates with BSA and collagen. Biocompatibility of diamond films was assessed by observing the metabolic activity of fibroblasts, osteoblasts, and MSCs. The role of ECM proteins in cell proliferation on diamond is investigated by measuring behavioral changes of adsorbed collagen.

### 3.1 Polycrystalline Diamond Coating on Acetabular Shells and Titanium Hemispheres

**Figure 1:**
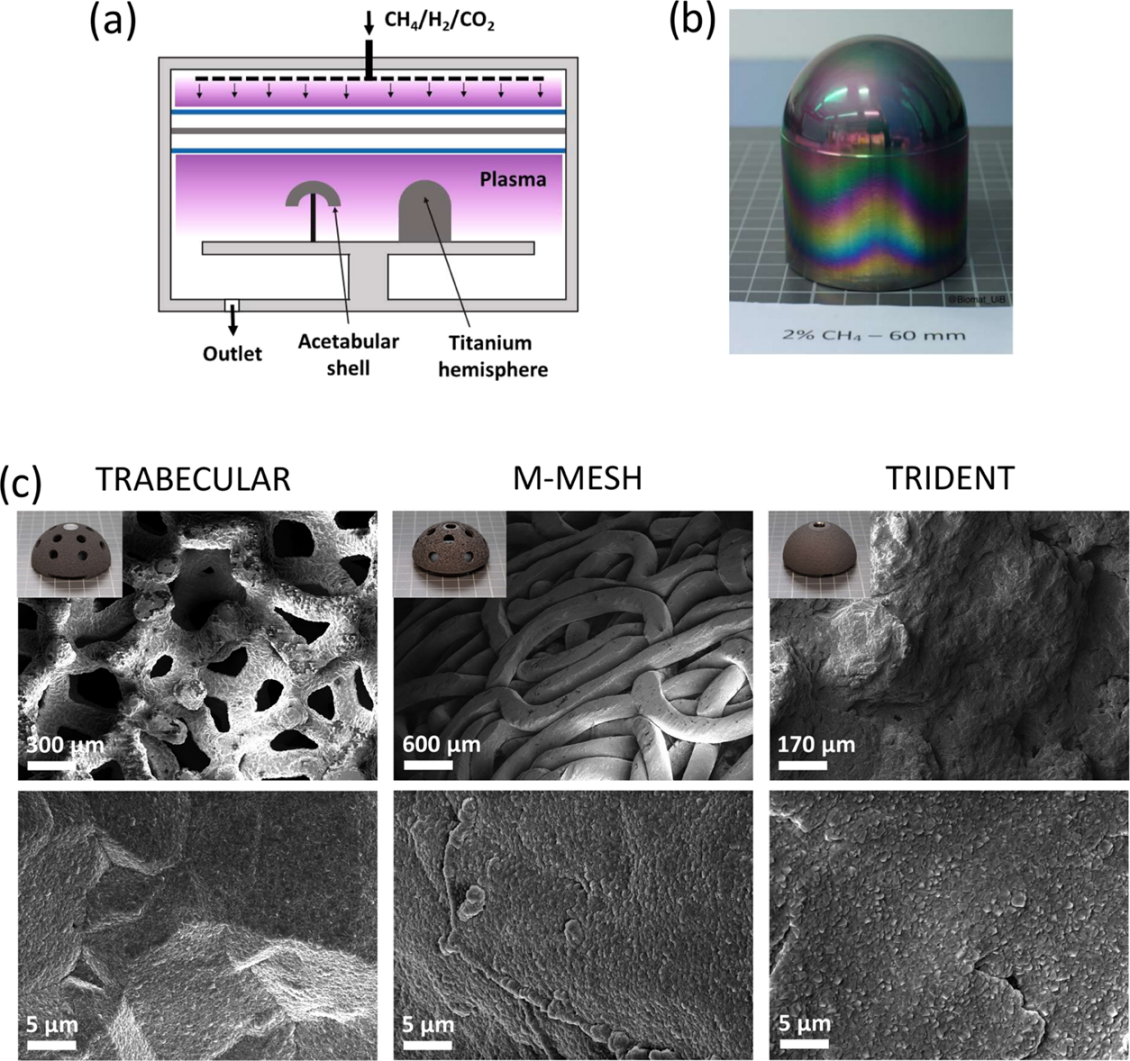
(a) Schematic drawing of the surface wave plasma chemical vapor deposition (SWP CVD) system. (b) Titanium hemisphere 60 mm in diameter coated with nanocrystalline diamond (NCD). (c) Scanning electron micrographs of NCD coating on TRABECULAR, M-MESH, and TRIDENT acetabular shells. The insets show photographs of the acetabular shells after CVD.

**Figure 2:**
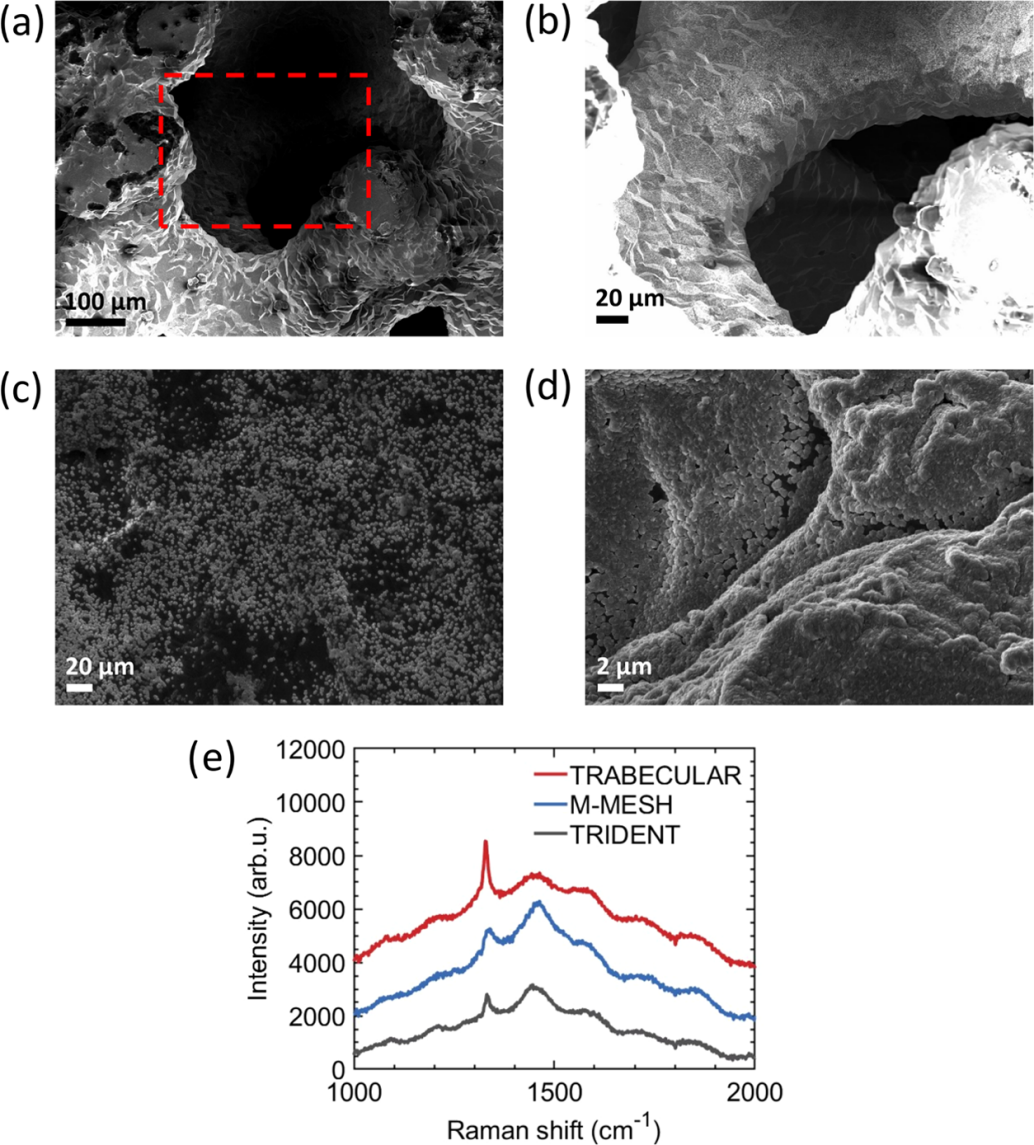
(a, b) Scanning electron microscopy (SEM) micrographs of the nanocrystalline diamond (NCD) coatings on the TRABECULAR acetabular shell. The dashed red line indicates area depicted in panel (b). (c) SEM micrographs of the NCD coating on the M-MESH acetabular shell, illustrating delayed nucleation and growth of diamond. (d) SEM micrographs of voids observed in the NCD coating on the TRIDENT acetabular shell. (e) Background-corrected Raman spectra of NCD films grown on acetabular shells.

Figure 1a shows a schematic drawing of the linear antenna SWP CVD system used to synthesize diamond at ∼400 °C. The titanium hemispheres (Figure 1b) and acetabular shells (Figure 1c) were placed within the so-called “CVD region”,^42,43^ which can extend up to 20 cm from the antennas, to achieve homogeneous CVD of diamond. Figure 1b shows a titanium hemisphere 60 mm in diameter coated with NCD. The thin-film interference pattern visible in the lower part of the sample indicates nonuniform thickness of the film extending up to the hemispherical part. Figure 1c shows SEM micrographs of uniform NCD coatings on TRABECULAR, M-MESH, and TRIDENT acetabular shells. The TRABECULAR mimics the structure of the trabecular bone and has up to 80% porosity with an average pore size of ∼400 μm. The porous structure of the shell makes seeding and, as a result, diamond CVD challenging. From SEM micrographs shown in Figure 2, we estimated that NCD was deposited on porous tantalum structures down to a depth of 600–800 μm. The reason for delayed nucleation and incomplete surface coverage deeper than 800 μm might be attributed not only to the lower ND seeding density but also to the reduced transport of precursor species into the pores. First porous layers act as barriers, which might prevent or limit the transport of atomic hydrogen and CH species, responsible for the growth of diamond,^42^ further. For M-MESH, the NCD film uniformly covers the surface of titanium fibers ∼300 μm in diameter except for some random regions of fibers intercrossing. The coatings in these regions do not fully cover the implant surface (see Figure 2c), which can be explained by the lower ND seeding density.

TRIDENT is an arc-deposited titanium shell with lower porosity and smaller pore size (30–100 μm) compared to the TRABECULAR shell. The NCD coating on TRIDENT is smooth and covers most of the investigated surface area except for a few random voids present in regions of high granularity seen in Figure 2d, which can be attributed to the variations of the seeding density due to the size of the pores and surface roughness.

Figure 2e shows the background-corrected Raman spectra of NCD films grown on TRABECULAR, M-MESH, and TRIDENT acetabular shells. The Raman spectra were collected at the poles of the shells. The characteristic diamond peak (D band) is observed at 1332 cm^–1^, and a broad line shape (G band) is visible at around 1580 cm^–1^. The broad peaks clearly visible near 1190 cm^–1^ and at around 1480 cm^–1^ are assigned to transpolyacetylene segments at grain boundaries and represent a signature of NCD.^44−46^ The amount of sp^3^-bonded carbon in NCD coatings is estimated to be 49, 36, and 44% for TRABECULAR, M-MESH, and TRIDENT, respectively, using the method detailed in ref (17).^17^

**Figure 3:**
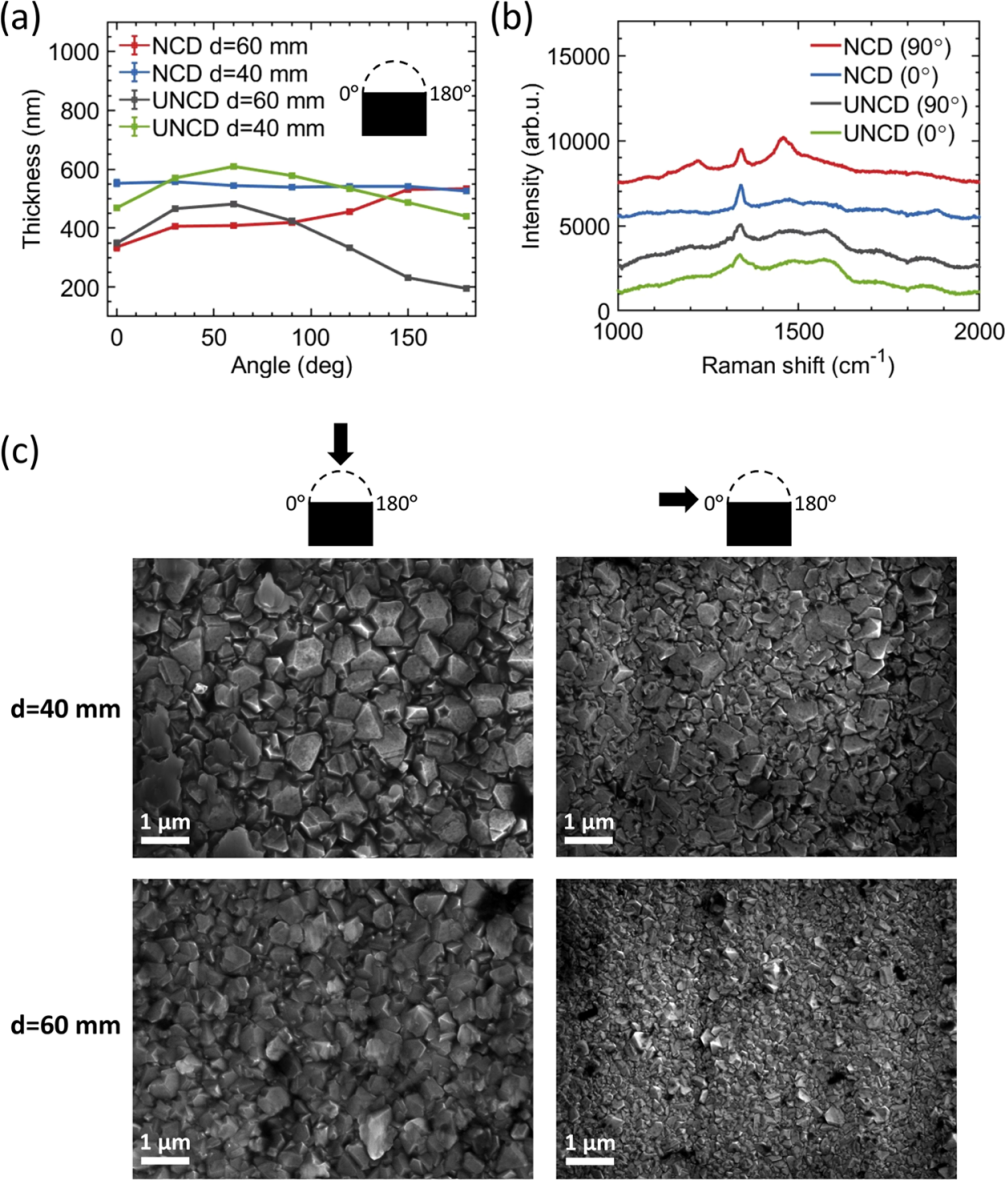
(a) Thickness profiles of nanocrystalline diamond (NCD) and ultrananocrystalline diamond (UNCD) films grown on titanium hemispheres. (b) Background-corrected Raman spectra of NCD and UNCD coatings on titanium hemispheres 60 mm in diameter. (c) Scanning electron microscopy (SEM) micrographs of NCD films on titanium hemispheres showing granularity of coatings at 0 and 90°.

The uniformity of diamond coatings was investigated on titanium hemispheres since thickness measurements of thin films on porous surfaces are challenging. Figure 3a shows thickness profiles along titanium hemispheres 60 and 40 mm in diameter for NCD and UNCD films, while the corresponding background-corrected Raman spectra are depicted in Figure 3b. The mean thickness for the 40 mm in diameter hemisphere is 543 nm (526 nm) for NCD (UNCD) and 441 nm (355 nm) for the 60 mm counterpart for NCD (UNCD). The uniformity of NCD on the 40 mm in diameter hemisphere is 2.8% and drops to 22.3% with the diameter increased up to 60 mm. The poor uniformity of UNCD coatings can be attributed to the drift of the surface waves on the linear antenna during the CVD process. This hypothesis is supported by the similarity of thickness profiles for both hemispheres (40 and 60 mm in diameter) coated with UNCD, hence indicating a systematic effect. We observed that the granularity of NCD films changes with distance from the top (at 90°, see Figure 3c) to the bottom (at 0°, see Figure 3c) of the hemispheres: for the 40 mm in diameter hemisphere, the average grain size decreases from 334 ± 46 to 241 ± 59 nm, while for 60 mm, it decreases from 265 ± 42 to 135 ± 31 nm. This can be explained by the decreasing plasma density with the distance from the antenna:^43^ increasing the diameter of the hemisphere increases the difference in plasma density at the top and at the bottom of the hemisphere, thus yielding a larger difference in the average grain size.

The uniformity of coatings on porous meshes might be improved by improving the uniformity and density of preseeding of the substrates. One possible option for nucleation enhancement would be to use adamantane seeding instead of ND as suggested by Tsugawa et al.^47^ Another possibility comes from Tsugawa et al.^23^ study, where they observed that the diamond nucleation rate increases with decreasing substrate temperature and suggested that under certain conditions, diamond nucleation takes place in the gas phase. In this way, nucleated diamond in plasma could diffuse toward the substrate and penetrate inside porous structures, finally precipitating on them and yielding a more uniform coating.

We observed that increasing the diameter of a titanium hemisphere from 40 to 60 mm resulted in a 10-fold increase in nonuniformity of NCD films. Since the diameters of TRABECULAR, M-MESH, and TRIDENT acetabular shells are close to 60 mm, we expect coatings uniformity to be ∼20%. Nonuniform and morphologically heterogeneous diamond coatings on orthopedic implants might have different tribological properties, which, as a result, might affect the integration and lifetime of implants. Therefore, the uniformity and structure of diamond coatings should have a homogeneous character. One possibility to improve the uniformity of the coatings is to position linear antennas in a way so they follow the curvature of the implant. However, this approach would require a customized LA CVD system.

### 3.2 BSA and Collagen Adsorption on NCD and UNCD Coatings

**Figure 4:**
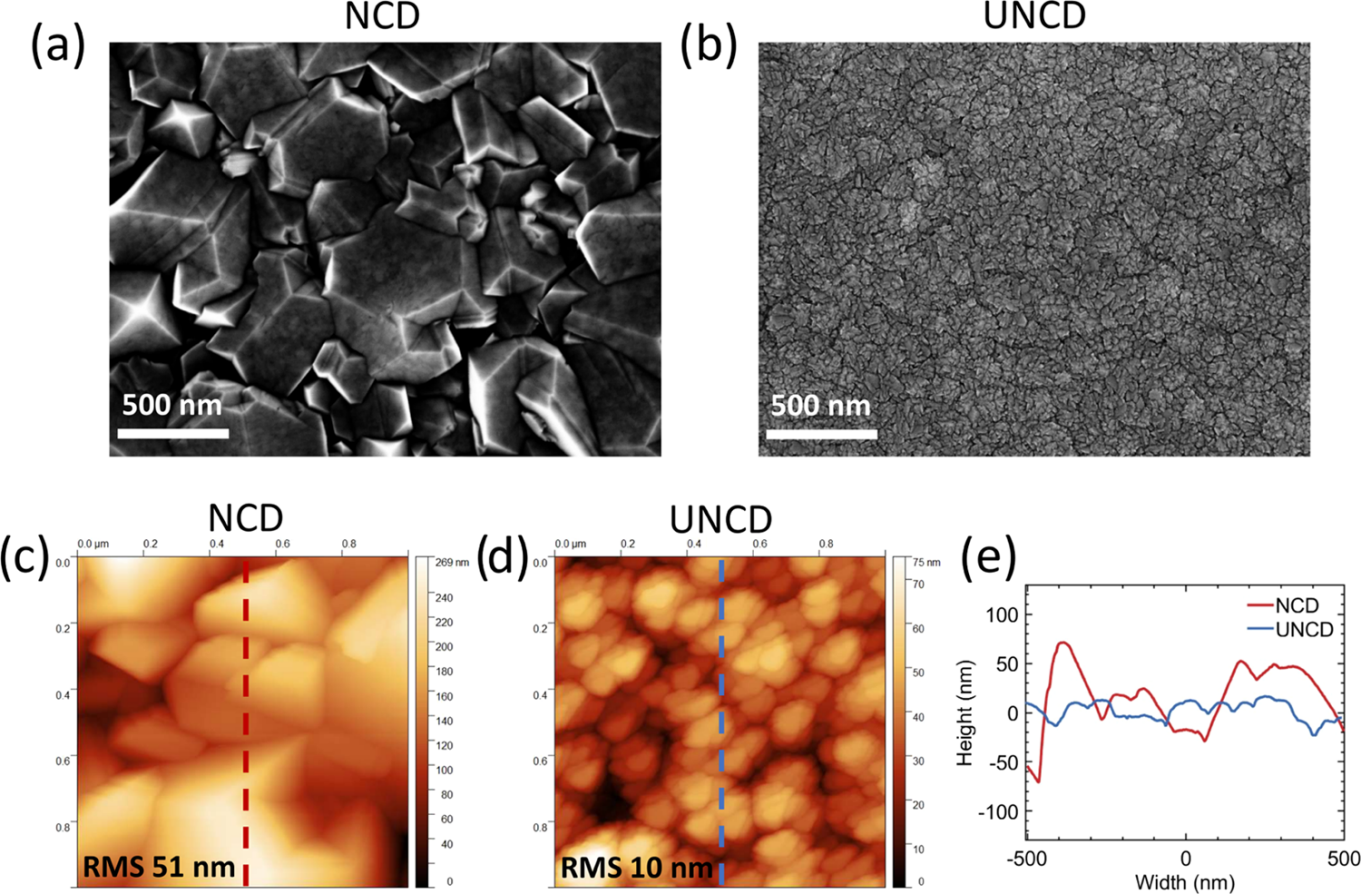
Scanning electron microscopy (SEM) micrographs of (a) nanocrystalline diamond (NCD) and (b) ultrananocrystalline diamond (UNCD) films on silicon wafers. High-resolution atomic force microscopy images of (c) NCD and (d) UNCD films’ topology on silicon substrates. (e) 1D profiles of surface topology scans shown as dashed red and dashed blue lines in panels (c) and (d), respectively.

We investigated the adsorption of proteins on NCD and UNCD films grown on silicon substrates using the same conditions as for the synthesis of diamond on acetabular shells and titanium hemispheres (Figure S1, Supporting Information). Figure 4a,b shows SEM micrographs of NCD and UNCD coatings, while Figure 4c,d depicts high-resolution AFM images of NCD and UNCD films’ topology on silicon substrates, respectively. The root-mean-square (RMS) roughness of the surface was measured to be 51 nm (10 nm) for NCD (UNCD) films. Figure 4e shows one-dimensional (1D) profiles of surface topology scans depicted in Figure 4c,d, indicating fivefold difference between roughness of NCD and UNCD films tested. The contact angle indicating wettability of the surfaces was measured to be 70.4 ± 3.0 and 64.9 ± 3.1° for hydrogenated NCD (NCD-H) and UNCD (UNCD-H), respectively, as well as 9.9 ± 0.5 and 11.0 ± 3.4° for oxygenated NCD (NCD-O) and UNCD (UNCD-O), respectively.

**Figure 5:**
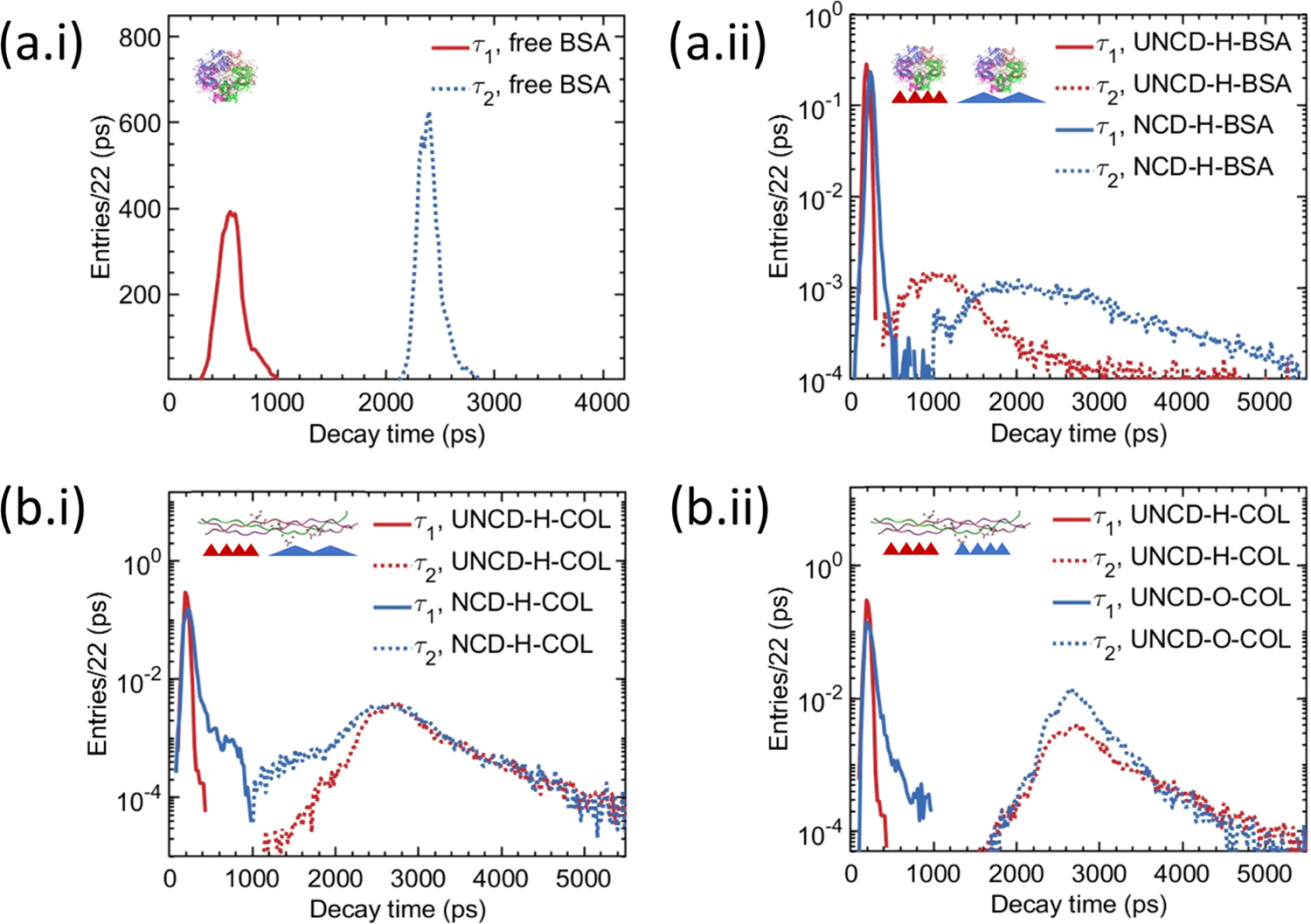
(a.i) Weighted histograms of fluorescence lifetimes τ_1_ and τ_2_ of bovine serum albumin and fluorescein isothiocyanate (BSA^FITC^) conjugates in 10 mM Tris buffer at pH 7.4. (a.ii) Normalized and weighted histograms of fluorescence lifetimes τ_1_ and τ_2_ of BSA^FITC^ adsorbed on hydrogenated ultrananocrystalline diamond (UNCD) and nanocrystalline diamond (NCD) films. (b.i) Normalized and weighted histograms of fluorescence lifetimes τ_1_ and τ_2_ of collagen fluorescein isothiocyanate (COL^FITC^) conjugates adsorbed on hydrogenated UNCD and NCD films and on (b.ii) oxygenated UNCD and NCD films.

First, we investigated BSA^FITC^ conjugates in 10 mM Tris buffer at pH 7.4. Figure 5a.i shows weighted histograms of fluorescence lifetimes τ_1_ and τ_2_ obtained from the decay matrix (128 × 128 pixels). Each entry in the histogram τ_1,2_^i^ is weighted by the corresponding decay time fraction *a*_1,2_^i^ extracted from the fit to a decay trace for a given pixel in the decay matrix. Both distributions are normally distributed and yield mean lifetimes of τ_1_ = 0.59 ns and τ_2_ = 2.42 ns. The fluorescence lifetime of fluorescein reported in the literature τ_FITC_ = 3.7–4.1 ns^48^ is longer compared to the longest obtained lifetime τ_2_. The shorter decay lifetime of BSA^FITC^ conjugates can be explained by dynamic self-quenching of the excited fluorophore in the encounter complex with monomers in the ground state, accelerated by fluorescence resonance energy transfer (FRET).^48^ Furthermore, FITC is bound to BSA through the ε-amino group of lysines of the albumin with 7–12 fluorophores decorating each protein. High labeling ratios yield shorter dye-to-dye distances and hence shortening of average lifetimes.^49^ We found from the goodness-of-fit that two lifetime components are sufficient to describe fluorescence decay of BSA^FITC^ conjugates. The longer lifetime (τ_2_) was attributed to the outermost fluorophores on albumin, while intermediate and locally concentrated FITC-FITC pairs were assigned to the shorter lifetime (τ_1_).

Figure 5a.ii shows normalized and weighted histograms of fluorescence lifetimes τ_1_ and τ_2_ of BSA^FITC^ conjugates adsorbed on UNCD-H and NCD-H films. The distributions of τ_2_ are centered at around 1 ns and 2 ns for UNCD-H and NCD-H, respectively, and have broader line shapes compared to τ_2_ distribution for albumin in a solution. The mean value of τ_1_ is 0.21 ns for UNCD-H and 0.25 ns for NCD-H. Since BSA undergoes irreversible structural changes upon adsorption on the hydrophobic surface,^50^ the broadening of τ_2_ distributions can be attributed to changes in distances of the outermost fluorophores relative to each other and relative to fluorophores associated to τ_1_. On the UNCD-H (NCD-H) surface, the difference in electronegativity between hydrogen (2.1) and carbon (2.5) produces H–C dipoles with +0.05*e* at the surface of hydrogen, yielding effective electric surface charge density of up to 1 × 10^14^ cm^–1^.^51^ The H–C dipoles provide sites for negatively ionized residues (ASP and GLU) of albumin with the total charge of approximately −9*e* at pH 7.2,^52^ hence inducing conformational changes, which in turn affects excitation lifetimes. Our findings of τ_2_ shortening for a smoother UNCD-H surface compared to the NCD-H surface agrees with results from Handschuh-Wang et al.,^48^ showing that the fluorescence lifetime of BSA^FITC^ adsorbed on polycrystalline diamond decreases with decreasing diamond grain size. The shortening of τ_1_ is attributed to surface-induced fluorescence quenching of fluorophores interacting with the diamond surface and changes in dye-to-dye distance due to conformational changes of BSA. These results show that the topology and hydrophobicity of the diamond surface can be used to affect conformational behavior of BSA upon adsorption. Since albumin is one of the most abundant proteins and adsorb immediately after implantation from blood and biological fluids, control over albumin adsorption on diamond coating might be used to tailor the integration of orthopedic implants.

Figure 5b.i shows normalized and weighted histograms of fluorescence lifetimes τ_1_ and τ_2_ of COL^FITC^ conjugates adsorbed on UNCD-H and NCD-H films. The distributions of τ_2_ have broad line shapes and are centered at around 2.6 ns. Collagen is decorated on average with one fluorophore, which yields longer dye-to-dye distances compared to BSA^FITC^ conjugates and hence longer lifetimes of τ_2_. Collagen at pH 7.2 assembles into fibrillar structures, typically forming a confluent monolayer on the substrate.^53^ Therefore, fluorophores can be distributed radially and be exposed to a solution, a diamond surface, or to neighboring amino acids. We attribute lifetime τ_2_ to fluorophore interaction with the solution and neighboring proteins. Similarly, as for BSA^FITC^, lifetime τ_1_ is attributed to surface-induced fluorescence quenching of fluorophores interacting with the diamond surface. The distribution of lifetime τ_1_ for NCD-H has a broader right-hand side tail compared to one for UNCD-H. Broadening of the distributions can be explained by topological differences between the two surfaces and the size of the collagen. Collagen consists of tropocollagen molecules ∼300 nm in length with diameters of ∼1.5 nm, leading to a high aspect ratio of ∼190.^54^ Higher roughness of the NCD-H surface (see Figure 4) given the length of collagen might increase the distance between fluorophores, residing on the fibrils and the surface, thus reducing fluorescence quenching and yielding broader distributions.

Figure 5b.ii shows distributions of lifetimes τ_1_ and τ_2_ of COL^FITC^ conjugates adsorbed on UNCD-H and UNCD-O films. The distributions of τ_2_ have similar line shapes and are centered at around 2.6 ns. The right-hand side tail of τ_1_ distribution for UNCD-O is broader compared to one for UNCD-H and cannot be a result of surface topology of the substrates. Cole et al.^55^ investigated the adsorption of a collagen fragment on the hydrogen-terminated and natively oxidized silicon surface using all-atom molecular dynamics. They found that within 5 ns, collagen might be highly mobile on the hydrophilic surface, while on the hydrophobic surface, it remains adsorbed more stably and maintains its helical structure. Therefore, we attributed the broadening of τ_1_ distribution for UNCD-O to the higher mobility of collagen on the hydrophilic diamond surface. These results show that adsorption of collagen on the diamond surface is affected by surface topology and wettability. Since collagen constitutes the major component of ECM and can promote adhesion and proliferation of MSCs,^56^ control of collagen adsorption by tailoring the diamond surface and chemistry might be beneficial for enabling better integration of implants into existing bone via stem cell recruitment and bone regeneration.^6^ Our findings indicate that the UNCD-H surface is best suited for tailoring collagen adsorption on diamond compared to other investigated surface topologies and chemistries.

### 3.3 Cell Attachment and Proliferation

**Figure 6:**
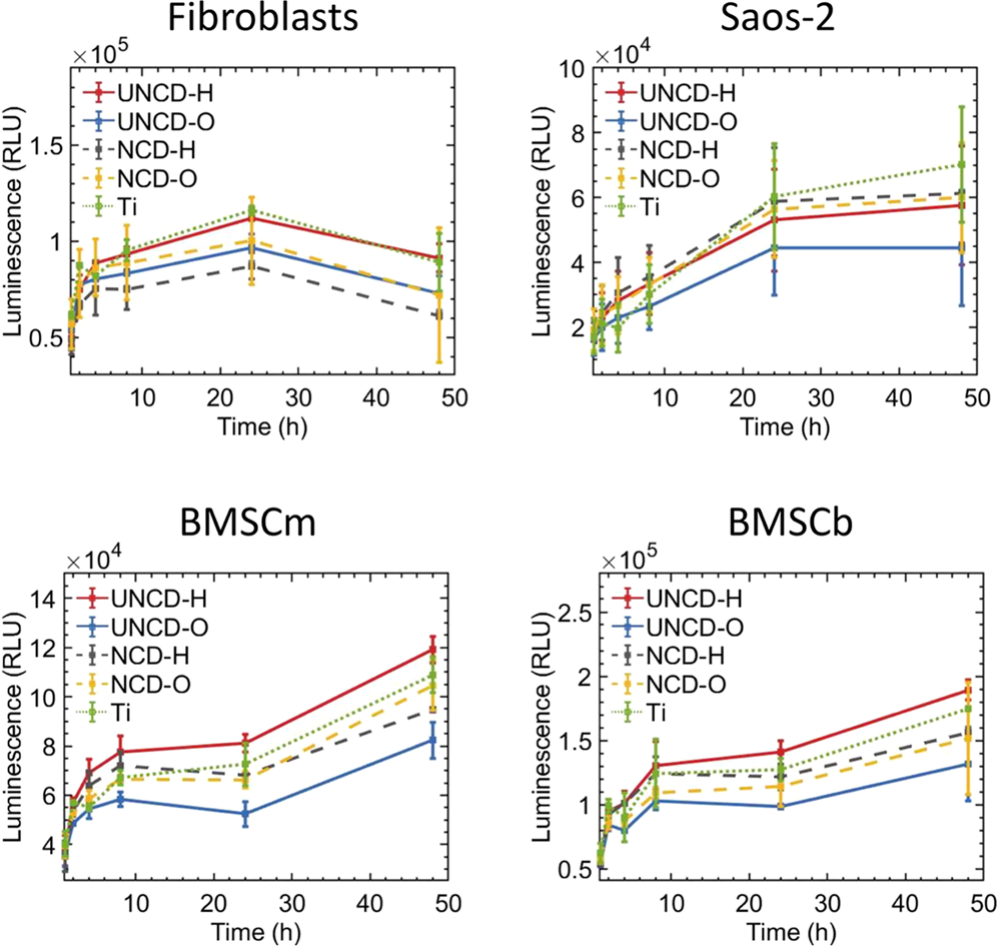
Growth of fibroblasts, osteogenic cells (Saos-2), and bone-marrow-derived mesenchymal stem cells (BMSCs) on titanium and diamond-coated substrates during the first 48 h of culture. Evolution in luminescence signal as a measurement of increasing metabolic activity.

Next, we sought to investigate the interaction and growth of primary adult fibroblasts, the osteogenic cell line Saos-2, and BMSCs, from two different donors (referred here as BMSCm and BMSCb), onto diamond films and bare titanium as the reference material. Figure 6 shows the evolution in luminescence signal as a measurement of increasing metabolic activity, which includes cell growth and division, of living cells over time. We observed that UNCD-H outperformed its oxygen-terminated counterpart and NCD films regarding the support of cell growth of fibroblasts and BMSCs over the whole 48 h incubation time. In addition, fibroblasts and BMSCs seeded on UNCD-H films exhibited growth profiles close to or slightly better than those observed on titanium (Figure 6). For Saos-2, all diamond films, except UNCD-O, performed equally well and close to the growth profile seen on titanium (Figure 6). Notably, metabolic activity during the first 4 h of culture, which reflects cell attachment, of BMSCs and Saos-2 was considerably higher on hydrogen-terminated UNCD and NCD films compared to oxygen-terminated counterparts and even to titanium (see Figure S2, Supporting Information, showing fold-change in the evolution of luminescence signal). This suggests that hydrogen-terminated diamond films facilitate the adsorption of cell attachment factors (e.g., ECM proteins like collagen) present in the cell culture medium and secreted by the cells. For fibroblasts, the initial cell attachment process was similar on all substrates except for NCD-H, on which fibroblasts attached poorly.

**Figure 7:**
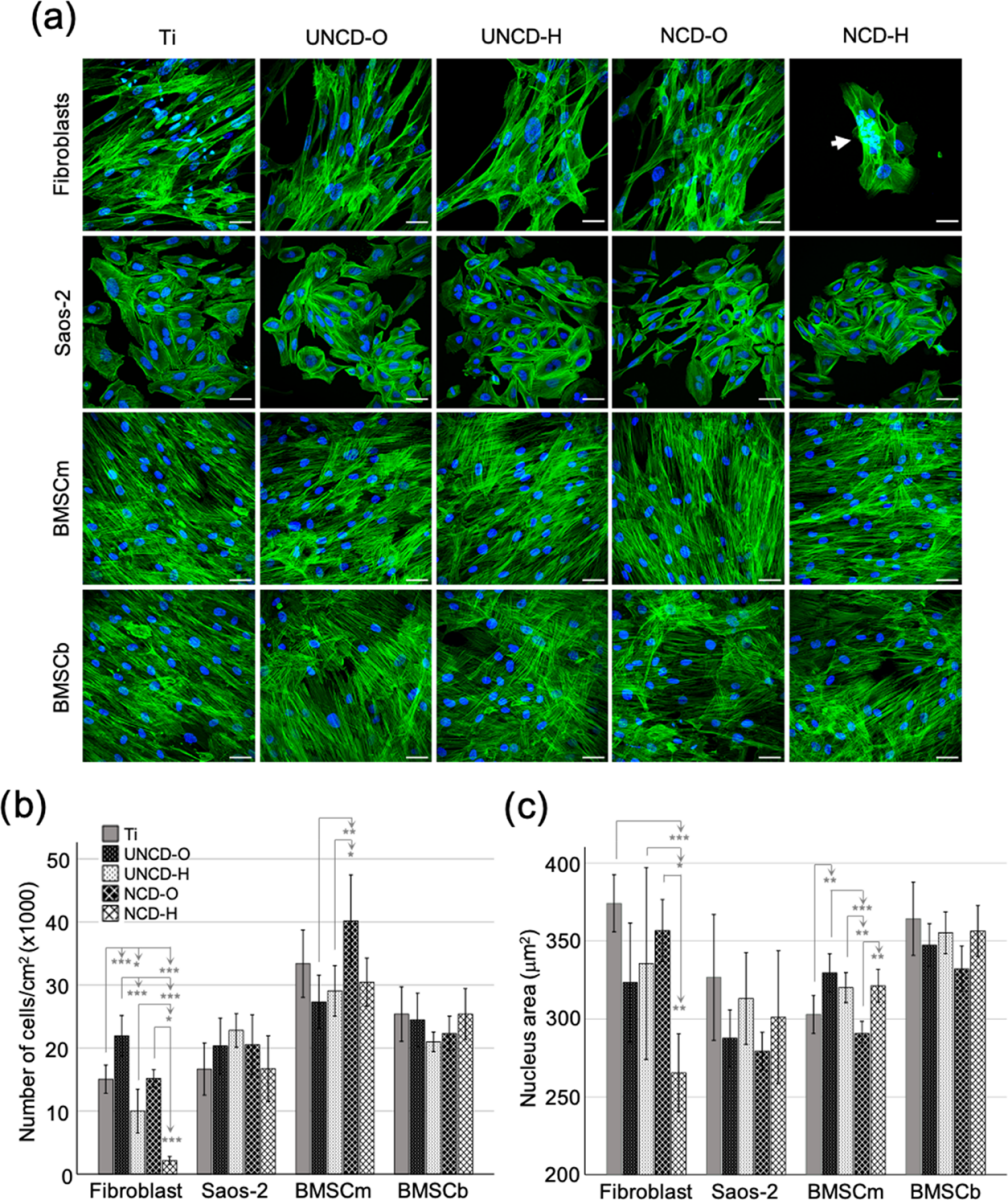
Growth of fibroblasts, Saos-2, and BMSCs on titanium and diamond-coated substrates after 5 days of culture. (a) Fluorescence micrographs of cells fixed at day 5 and stained with phalloidin-ATTO 565 (green) and DAPI (blue) to visualize actin filaments (F-actin) and nuclei, respectively. Shown are maximum *z*-projections of merged phalloidin-ATTO 565/DAPI. Arrow points to a densely packed cell cluster. Scale bars are 50 μm. (b) Number of cells per cm^2^ and (c) nucleus area at day 5 of culture. Statistical annotations: ′*′ 0.05 > *p* > 0.01, ′**′ 0.01 > *p* > 0.001, ′***′ *p* < 0.001.

To further validate these observations, we carried out a microscopic analysis of the three cell types cultured for 5 days on diamond-coated films and titanium. As can be seen in Figure 7, BMSCs seemed to grow equally well on all surfaces after 5 days of culture. The BMSCs were able to form confluent monolayers of elongated cells with fully developed filamentous (F) actin bundles (i.e., stress fibers) across the cytoplasm (Figure 7a). The number of BMSCm present at day 5 was higher on NCD-O than on any of the other substrates (Figure 7b), although the difference was only significant when comparing NCD-O (40 ± 7 × 10^3^ cells/cm^2^) with UNCD substrates (27 ± 7 × 10^3^ cells/cm^2^ on UNCD-O and 29 ± 6 × 10^3^ cells/cm^2^ on UNCD-H). Furthermore, we analyzed the nucleus area (Figure 7c) and nucleus aspect ratio (Figure S3, Supporting Information) as indicators of cell expansion and shape, respectively. We used these parameters to infer the extent of support provided for cell attachment by the different substrates. The larger nucleus area yields a larger cell’s cytoskeleton expansion and thus better attachment. In the case of full coverage of the surface (i.e., high cell confluency), as observed for BMSCs, the nucleus area was inversely proportional to the cell number. We observed that BMSCs cultured on NCD-O developed significantly smaller nuclei than on the other substrates except for titanium, where the dimensions were similar (303 ± 6 μm^2^ for Ti and 291 ± 6 μm^2^ for NCD-O). Interestingly, BMSCm cultured on titanium displayed nuclei with a larger aspect ratio compared with cells on diamond-coated substrates, i.e., higher cell elongation on titanium than on diamond coatings. This cannot be solely attributed to the dense cell packing due to confluency since we observed a higher number of cells on NCD-O than on titanium. The cell number, nucleus area, and nucleus aspect ratio values for BMSCs from the BMSCb donor were comparable among all substrates (see Figure 7).

The surface coverage by the Saos-2 cell population was slightly larger on UNCD-H than on the other substrates (Figure 7a,b). However, no statistically significant differences were obtained while comparing the number of cells per cm^2^ on each substrate. Furthermore, the osteogenic Saos-2 cells appeared smaller in size on NCD films and on UNCD-O than on UNCD-H and on titanium. Analysis of nuclei areas revealed no statistically significant differences while comparing means and medians between different substrates (Figure 7c). However, the size distribution of nuclei areas for Saos-2 on NCD-O and UNCD-O films is considerably narrower than on the other substrates, with mean and median values below 290 μm^2^. In contrast, the mean nucleus area of Saos-2 cultured on titanium was 327 ± 20 μm^2^. This suggests better cell attachment and expansion of Saos-2 on titanium and UNCD-H than on oxygen-terminated diamond films counterparts. In addition, Saos-2 cells developed a polygonal shape with abundant F-actin bundles along the cell periphery on titanium, UNCD films, and NCD-H. Meanwhile, Saos-2 exhibited an oval to spindle-like shape when cultured on NCD-O. These observations were further corroborated by the analysis of nuclei aspect ratio (Figure S3, Supporting Information). The Saos-2 cells cultured on NCD-O displayed elongated nuclei with a mean aspect ratio value of 1.77 ± 0.05, whereas on titanium, the value was significantly smaller (1.54 ± 0.03, *p* < 0.001).

We observed that cell confluency for fibroblasts was higher on titanium and UNCD-O compared with other diamond films, with the poorest attachment observed for hydrogenated surfaces (Figure 7). Indeed, the number of cells on UNCD-O at day 5 was significantly higher (*p* < 0.001) than on titanium (22 ± 3 × 10^3^ and 15 ± 2 × 10^3^ cells/cm^2^, respectively) and on hydrogen-terminated films (10 ± 3 × 10^3^ cells/cm^2^ on UNCD-H and 2 ± 1 × 10^3^ cells/cm^2^ on NCD-H). On NCD-H, cells tended to grow into highly packed cell clusters, i.e., colonies, rather than single, spindle-shaped cells with long F-actin bundles across the cytoplasm as observed on titanium and oxygen-terminated films. These observations were further validated with the analysis of nuclei areas (see Figure 7c). Fibroblasts cultured on NCD-H developed significantly smaller nuclei compared to those on titanium and on NCD-O (266 ± 13, 374 ± 9, and 357 ± 10 μm^2^, respectively). Nuclei of fibroblast on UNCD films were also smaller than on titanium, although not statistically significant (323 ± 19 μm^2^ on UNCD-O and 336 ± 31 μm^2^ on UNCD-H). Furthermore, fibroblasts on NCD-H displayed a wide distribution of nucleus aspect ratio values ranging from 1.23 to 2.57 (1.82 ± 0.11), whereas on titanium and on UNCD-H, the value ranges were 1.55–1.96 (1.78 ± 0.04) and 1.47–1.75 (1.71 ± 0.04), respectively (Figure S3, Supporting Information). These data show that fibroblasts on hydrogen-terminated diamond were rather small compared to fibroblasts cultured on titanium, indicating poor cell attachment.

**Figure 8:**
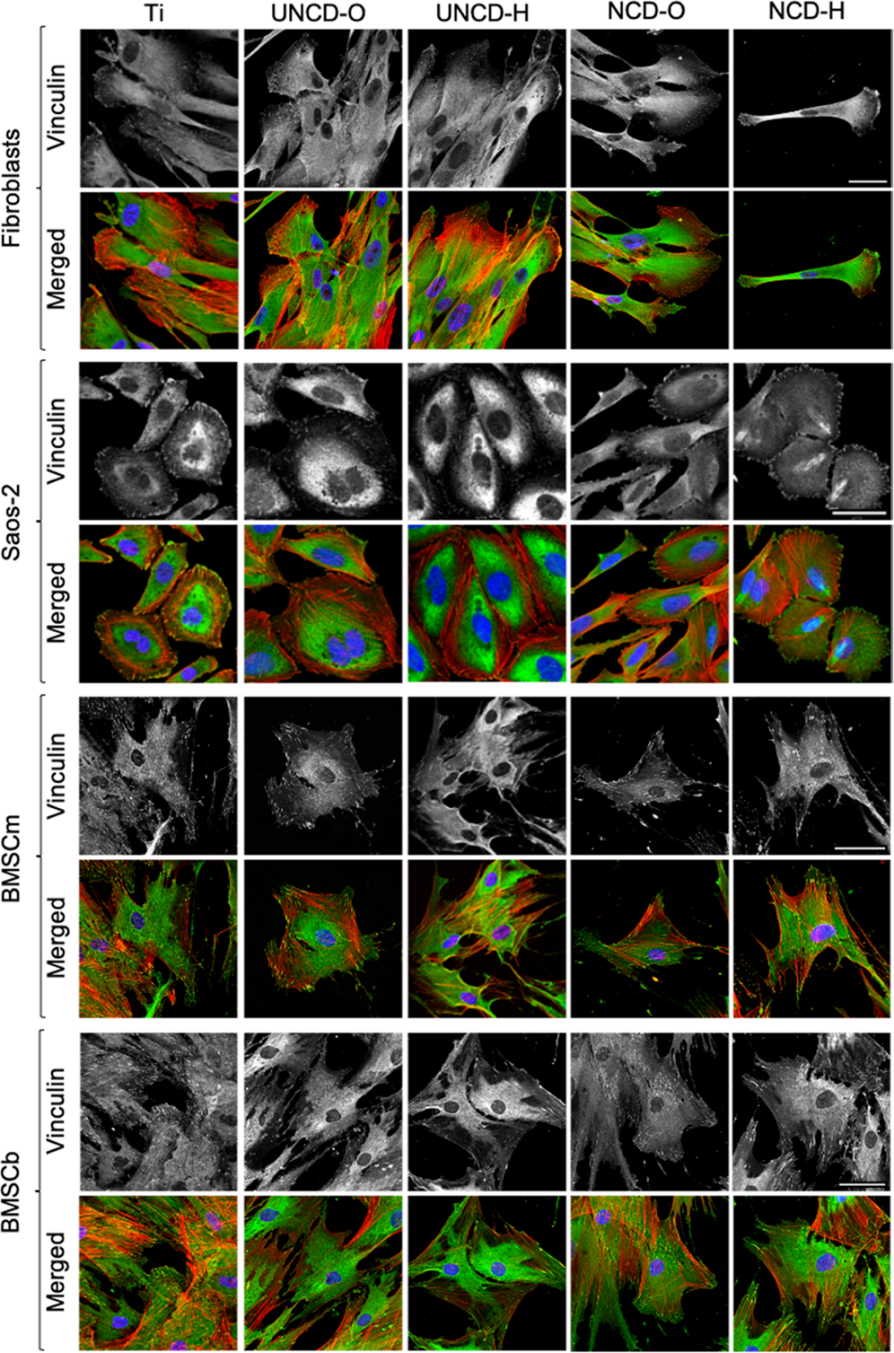
Fluorescence micrographs of cells growing on titanium and diamond films. Cells were fixed at day 5 and immunostained for vinculin (green) and counterstained with phalloidin-ATTO 565 (red) and DAPI (blue) to visualize the actin filaments (F-actin) and nuclei, respectively. Shown are maximum *z*-projections of merged and single vinculin channels. Scale bars are 50 μm.

Next, we examined whether focal adhesion (FA) assembly would differ between substrates as an additional indicator of cell–substrate interaction and attachment since FAs are molecular assemblies that connect cells to the ECM deposited on the underlying substrate. For visualization of FAs, cells were immunostained to detect vinculin, an integral cytoskeletal protein of FA that links F-actin to the membrane at sites of cell–substrate anchoring. Immunostaining in BMSCs revealed diffused staining through the cytoplasm and bright patches of vinculin at converging sites of F-actin bundles with the membrane at the basal side of the cells cultured on all substrates, with no apparent distinction between substrates as seen in Figure 8. All substrates supported, to a similar extent, the formation of FA and thus anchoring of BMSCs to the surface. For Saos-2 cells, vinculin staining showed an assembly of FA at F-actin vertices in the basal side of the cell, particularly at the cell’s periphery (Figure 8). Cytoskeleton organization and FA spatial distribution were comparable for cells plated on titanium and UNCD films. However, cells cultured on NCD films displayed divergent features. As mentioned above, cells cultured on NCD-O exhibited two main morphologies: spindle-like and polygonal. The vinculin staining in spindle-like Saos-2 was rather diffused throughout the cell body with no clear accumulation at F-actin vertices, suggesting the inefficient formation of FA. Polygonal-shaped Saos-2 on NCD-O displayed similar cytoskeleton organization, including FAs, as observed on titanium and UNCD films. Saos-2 cultured on NCD-H developed a polygonal shape with bright vinculin patches at F-actin vertices in the cell’s periphery and at the poles and across the dorsal surface of the nucleus in polarized cells. FAs in the proximity of the nucleus are associated with the actin cap and are bigger in size and more elongated than other ventral FAs.^57^ In addition, FAs coupled to the actin cap are more sensitive to mechanotransduction than other FAs^57^ and the actin cap is associated with persistent cell migration.^58^ Therefore, Saos-2 cultured on NCD-H might be more migratory active than on the other substrates. It is important to note that FA assembly, maturation, and disassembly depend on mechanical tension generated, sensed, and transmitted by actomyosin contractibility. FAs are generally viewed as anchoring sites for stagnated cells. However, FAs are also essential for generation of traction forces during migration.^59^ Overall, attachment and growth of Saos-2 seemed to excel on titanium and UNCD-H. Finally, immunostaining of fibroblasts revealed diffuse cytoplasmic localization of vinculin with bright patches at F-actin vertices, particularly at lamellipodia in the leading edge of polarized, migratory cells (Figure 8). FAs seemed to be less abundant and weaker in fluorescence signal in fibroblasts cultured on hydrogenated films and on NCD-O than on titanium and on UNCD-O. Taken together, titanium and UNCD-O were advantageous for attachment and growth of fibroblasts, while hydrogen-terminated diamond films were not.

Fibroblasts are the primary source of ECM, which includes fibronectin, laminins, and collagen matrix. These matrix-producing cells can adhere to and grow on any of the aforementioned proteins. However, cell adhesion forces and proliferation rates are higher on fibronectin than on laminin and collagen.^60^ In addition, fibronectin adsorption is favored on hydrophilic surfaces while laminin and collagen adsorb better onto hydrophobic ones.^60^ Therefore, the poor cell growth observed for fibroblasts on hydrogen-terminated diamond films may be explained by low fibronectin adsorption and disruption of its active conformation, as shown by Baujard-Lamotte et al.,^61^ for adsorption of fibronectin on hydrophobic polystyrene. In contrast, Saos-2 and BMSCs grow preferentially on collagen. However, wettability alone cannot explain cell behavior since fibroblasts grew better on UNCD-H than on the NCD-H surface both of which have similar contact angle values (64.9 ± 3.1 and 70.4 ± 3.0°). Furthermore, surface roughness may also influence a fibronectin three-dimensional (3D) structure as is observed for BSA in this study. Overall, surface topology plays a pivotal role in cell adhesion, proliferation, migration, and differentiation determined by the ability of cells to form functional FA.^62^ Zhao et al.^63^ demonstrated that the attachment and proliferation of MG63 osteoblast-like cells were superior on flat nanostructures than on Ti disks with submicrometer features. Similarly, Hou et al.^64^ observed that MSCs cultured on surface roughness gradients, ranging from the nanometer to submicrometer scale, spread better on low- than on high-roughness surfaces. Results in ref^64^ and in this study indicate that the FA assembly, mechanotransduction, and ultimately MSC fate are influenced by the substrate surface topology and chemistry. Therefore, diamond film roughness and surface functionalization are factors that can be finely tuned to control cell fate.

Taken together, UNCD-H and NCD-H appear to be excellent candidate coatings for orthopedic implants since both support colonization of BMSCs and osteogenic cells as well as medical grade titanium. In addition, fibroblasts showed lower colonization on hydrogen-terminated diamond than on titanium. This may help prevent implant failure due to the development of fibrosis,^65,66^ which is driven by uncontrolled growth of fibroblasts and their transformation to myofibroblasts, leading to excess deposition of pathological ECM around the implant. In this regard, it has been shown that released metal particles generated by mechanical loading in metal-on-metal hip implants are able to activate synovial fibroblasts. This leads to abnormal deposition of ECM, fibrosis, and ultimately implant failure.^67^ Diamond coatings for metal-on-metal implants could prevent or minimize the release of metal and/or diamond wear particles due to its excellent resistance and wear properties. Even though diamond particles may be released, some studies suggest that diamond nano/microparticles have low cytotoxicity.^68,69^ Although promising, these results should be taken with care. Further in vitro analyses are needed to investigate growth and activation of synovial fibroblasts, ECM deposition, and release of wear particles from diamond-coated implants.

## 4 Conclusions

In this work, for the first time, we demonstrated deposition of NCD at low temperatures (∼400 °C) on three types of acetabular shells each having different surface structures and porosities, showing the high potential of the surface wave plasma CVD technique for coating orthopedic implants. Coatings on all acetabular shells uniformly covered high- and low-porosity structures present on the surface. We achieved diamond synthesis on porous tantalum structures, which mimic the structure of the trabecular bone. The entire surface of each shell was covered with NCD apart from random regions, where diamond films contained small voids or showed signs of delayed nucleation attributed to imperfect seeding density. To assess the uniformity of the coatings, NCD and UNCD films were also deposited on titanium hemispheres purposely chosen to mimic the shape of the acetabular shells. We demonstrated that the surface wave plasma CVD technique is suitable for achieving good uniformity (2.8%) NCD coatings on 40 mm in diameter titanium hemispheres. We found that increasing the diameter of a titanium hemisphere up to 60 mm significantly decreased the uniformity of diamond films. Furthermore, surface topology analysis revealed decreased granularity of the coatings with the distance from linear antennas due to the reduced plasma density.

The biocompatibility of the coatings was assessed by investigating the adsorption of albumin and type I collagen and monitoring in real time the proliferation of primary adult fibroblasts, osteogenic cells Saos-2, and bone-marrow-derived MSCs. By measuring fluorescence lifetimes, we studied the conformational changes of albumin, showing that the surface topology of diamond has a pronounced effect on the structure of adsorbed albumin. Results obtained for collagen indicate that the hydrophilicity of a diamond surface can yield higher mobility and reduced structural stability of collagen. Lastly, we found that hydrogen-terminated UNCD and NCD support the colonization of MSCs and osteogenic cells and diminish the colonization of fibroblasts compared to titanium. The proliferation of osteogenic cells on hydrogenated UNCD was found to be better than that on its oxygen-terminated counterpart, indicating a possible correlation with observed behavior of adsorbed collagen. Biocompatibility assessment shows that the surface topology and chemistry of diamond play a profound role in adsorption of proteins and cell proliferation. The results for hydrogenated diamond films show that this type of coating has great potential to be an excellent candidate for orthopedic implants.

## Supporting Information Available

Deconvoluted C 1s (carbon) high-resolution X-ray photoelectron spectra (XPS); fold-change in the evolution of luminescence signal vs titanium substrate; nuclei aspect ratio of cells at day 5 of culture on titanium and diamond films (PDF)
