# Supplementary material for: Polycrystalline Diamond Coating on Orthopedic Implants: Realization and Role of Surface Topology and Chemistry in Adsorption of Proteins and Cell Proliferation
Source: ACS Appl Mater Interfaces. 2022 Sep 22;14(39):44933–46. doi: 10.1021/acsami.2c10121 (PMC9542704; doi:10.1021/acsami.2c10121)
Supplement: Supplementary file 1 — am2c10121_si_001.pdf [file am2c10121_si_001.pdf]

Supporting Information

Polycrystalline Diamond Coating on Orthopaedic Implants: Realization, and Role of Surface Topology and Chemistry in Adsorption of Proteins and Cell Proliferation

Justas Zalieckas[1,], Ivan R. Mondragon[2], Paulius Pobedinskas[3,4], Arne S. Kristoffersen[1], Samih Mohamed-Ahmed[2], Cecilie Gjerde[2], Paul J. Høl[5,6], Geir Hallan[5,6], Ove N. Furnes[5,6], Mihaela Roxana Cimpan[2], Ken Haenen[3,4], Bodil Holst[1] and Martin M. Greve[1].*

1. Department of Physics and Technology, University of Bergen, Allegaten 55, Bergen, Norway

2. Department for Clinical Dentistry, University of Bergen, Årstadveien 19, Bergen, Norway

3. Institute for Materials Research (IMO), Hasselt University, Wetenschapspark 1, 3590 Diepenbeek, Belgium

4. IMOMEC, IMEC vzw, Wetenschapspark 1, 3590 Diepenbeek, Belgium

5. Department of Orthopaedic Surgery, Haukeland University Hospital, Jonas Lies vei 65, Bergen, Norway

6. Department of Clinical Medicine, University of Bergen, Jonas Lies vei 87, Bergen, Norway

*Corresponding author:

E-mail: justas.zalieckas@uib.no

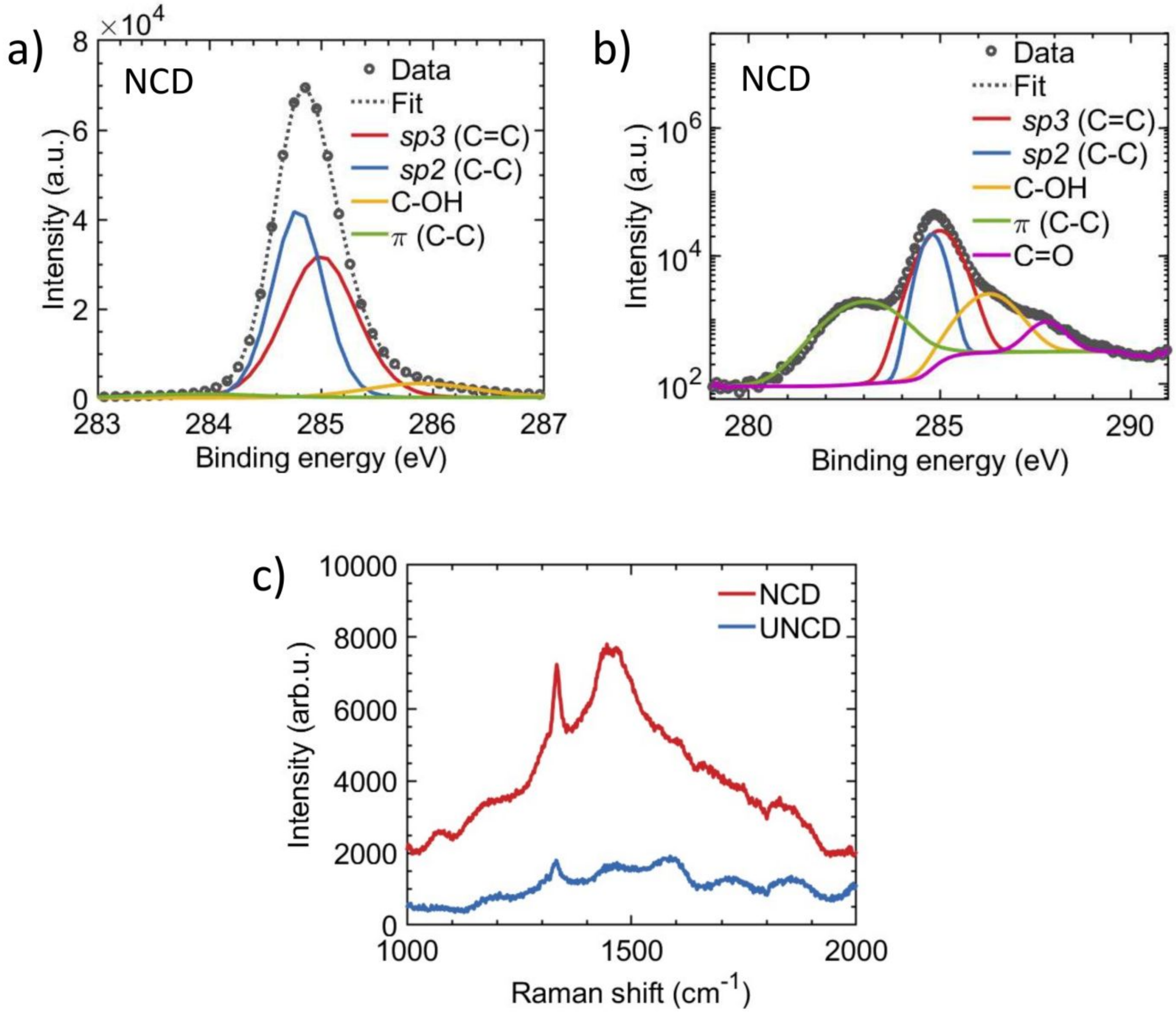

Figure S1. Deconvoluted C 1s (carbon) high-resolution X-ray photoelectron spectra (XPS) of (a) hydrogenated and (b) oxygenated nanocrystalline diamond (NCD) film showing peaks fitted for sp3, sp2, C‒OH, C=O bonds and π-bonded C atoms. (c) The background corrected Raman spectra of NCD and UNCD films on silicon wafers.

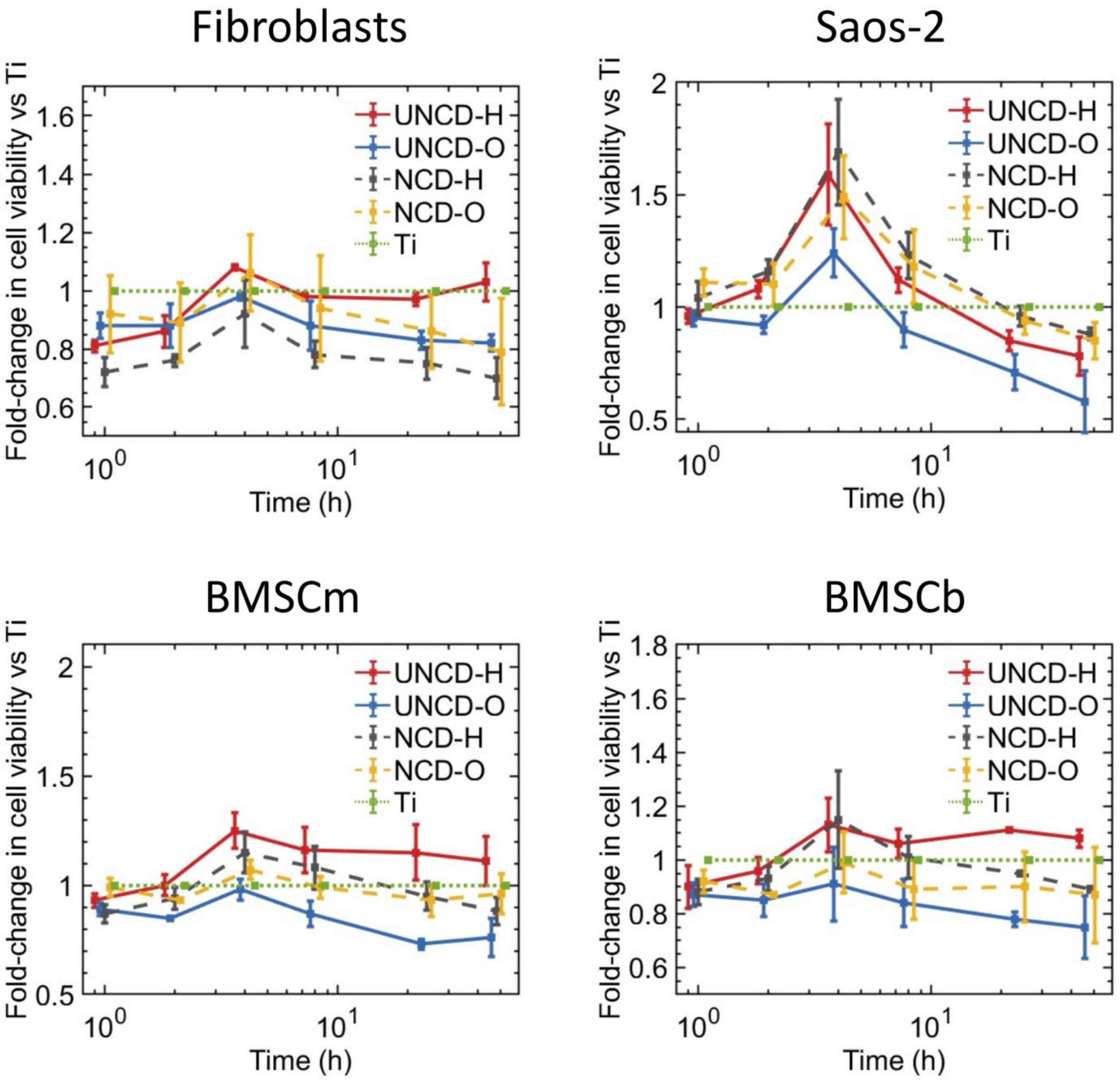

Figure S2. Fold-change in the evolution of luminescence signal versus titanium substrate for (a) fibroblasts, (b) osteosarcoma cell line (Saos-2), (c) human bone marrow-derived mesenchymal stem cells (BMSCm) and BMSCb. Data points are spread around nominal values for better representation of error bars.

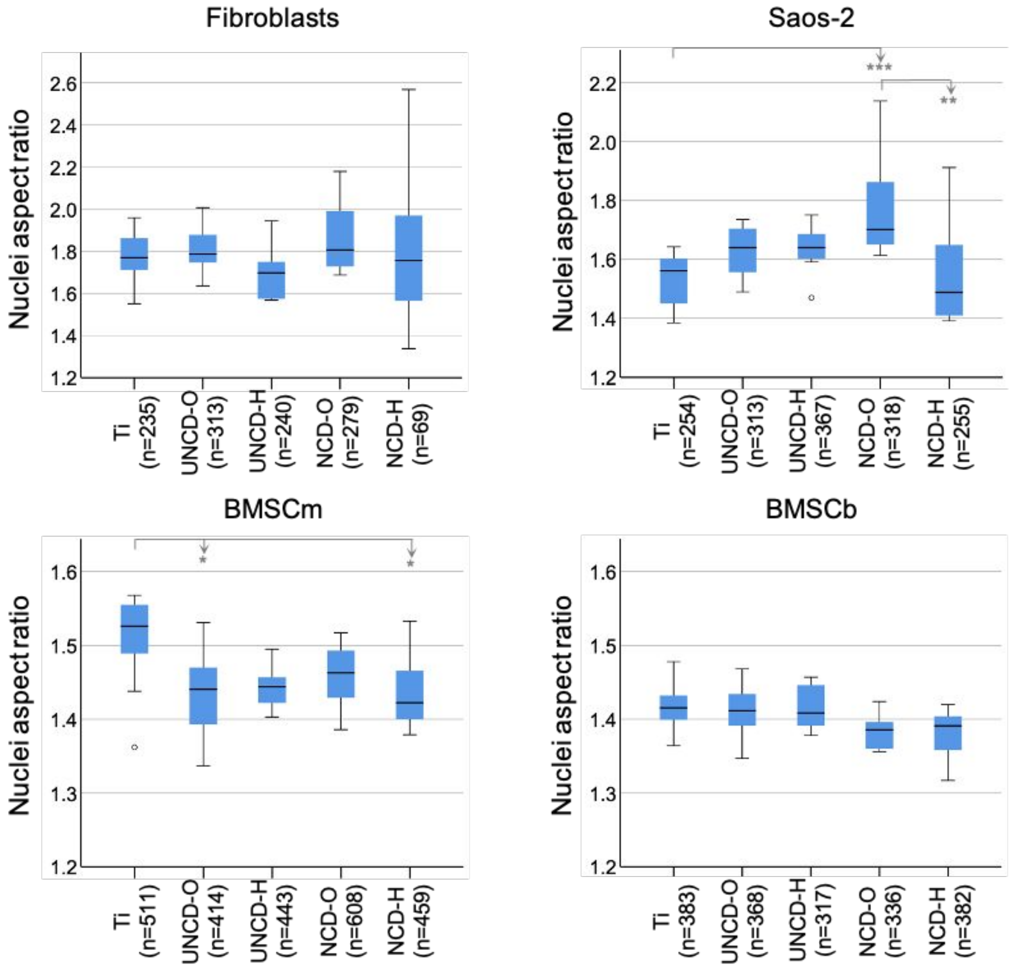

*Figure S3. Nuclei aspect ratio of cells at day 5 of culture on titanium and diamond films. The number of cells analyzed in each condition is enclosed in parenthesis. Statistical annotations: `*´ 0.05>p>0.01, `**´ 0.01>p>0.001, `***´ p<0.001.*
